# Implementing a Standardized Social Networks Testing Strategy in a Low HIV Prevalence Jurisdiction

**DOI:** 10.1007/s10461-018-2146-x

**Published:** 2018-05-15

**Authors:** Casey Schumann, Danielle Kahn, Michelle Broaddus, Jacob Dougherty, Megan Elderbrook, James Vergeront, Ryan Westergaard

**Affiliations:** 1 AIDS/HIV Program, Division of Public Health, Wisconsin Department of Health Services, Madison, WI USA; 2 Division of Infectious Diseases, Department of Medicine, University of Wisconsin School of Medicine and Public Health, Madison, WI USA; 3 Center for AIDS Intervention Research, Medical College of Wisconsin, Milwaukee, WI USA

**Keywords:** HIV testing, Social network, Counseling, Testing, Referral

## Abstract

Alternative HIV testing strategies are needed to engage individuals not reached by traditional clinical or non-clinical testing programs. A social networks recruitment strategy, in which people at risk for or living with HIV are enlisted and trained by community-based agencies to recruit individuals from their social, sexual, or drug-using networks for HIV testing, demonstrates higher positivity rates compared to other non-clinical recruitment strategies in some jurisdictions. During 2013–2015, a social networks testing protocol was implemented in Wisconsin to standardize an existing social networks testing program. Six community-based, non-clinical agencies with multiple sites throughout the state implemented the protocol over the 2-year period. Both quantitative and qualitative data were collected. The new positivity rate (0.49%) through social networks testing did not differ from that of traditional counseling, testing, and referral recruitment methods (0.48%). Although social networks testing did not yield a higher new positivity rate compared to other testing strategies, it proved to be successful at reaching high risk individuals who may not otherwise engage in HIV testing.

## Introduction

Undiagnosed HIV infection is estimated to account for one-third of HIV transmissions in the United States [1]. While the proportion of persons unaware of their infection has improved over time, it remains high among some populations. The Centers for Disease Control and Prevention (CDC) estimate that 15% of HIV-infected individuals are unaware of their infection; proportions of undiagnosed infection are greater among youth ages 13–24 and among racial and ethnic minorities compared to Whites [2]. More than 20% of individuals have progressed to AIDS by the time of HIV diagnosis, indicating that many individuals live for years with undiagnosed infection, creating opportunities for transmission [2].

HIV testing is a critical stage in the HIV care continuum, as it both identifies undiagnosed HIV, and is the necessary first step in linking infected individuals to care and treatment services. Research has shown that those at highest risk for HIV infection, such as young men who have sex with men (MSM), frequently have poor access or adherence to routine clinical care and therefore may benefit from more accessible and directed services [3, 4]. As such, the CDC provides financial support and guidance for non-clinical HIV counseling, testing, and referral (CTR) programs, whereby agencies define how their HIV testing services will be made accessible, appealing, appropriate, and culturally relevant to a defined target population. CTR programs then use a variety of methods to recruit members of that population to be tested [5].

Social networks recruitment is one of several possible recruitment strategies for CTR programs [5]. Social networks recruitment is a peer-based strategy in which people at high risk for or living with HIV (recruiters) are enlisted to recruit individuals from their social, sexual, and drug-using networks (network associates) for HIV testing [5]. Recruiters receive training and coaching on techniques to approach and motivate their peers to be tested for HIV, which typically involves monetary incentives for both the recruiter and the network associate (NA).

Previous studies suggest that SNT is an effective and efficient method for identifying persons with undiagnosed HIV infection [6–8]. In a CDC-funded demonstration project, 5.6% of NAs recruited for HIV testing through social networks recruitment were newly diagnosed with HIV infection; five-times higher than the national average for other CTR recruitment strategies [6]. Hailkitis et al. found SNT had a 3.6 greater odds for detecting HIV infection among Black MSM in New York City compared to alternative venue testing [7]. Besides the high positivity rate, SNT demonstrates success in reaching Black MSM who have never previously tested for HIV [9] as well as those reporting condomless intercourse [7].

The Wisconsin Division of Public Health (DPH) has supported SNT as a CTR recruitment strategy since 2008. The goals of Wisconsin’s SNT program were to improve detection of undiagnosed HIV infection and to increase access to testing among high-risk populations, such as Black MSM and people who inject drugs. The DPH provided guidance to SNT sites based on recommendations developed and disseminated by the CDC [6], but there was no written protocol, and thus no standards by which to measure agency adherence to the SNT model or for agencies to assess their own efforts. Agencies also did not have staff dedicated to SNT and therefore were not spending as much time on recruitment and coaching as the SNT model intends. Finally, some recruiters were identifying large numbers of NAs who were not at high risk of acquiring HIV. These implementation challenges were temporally associated with a decrease in the HIV positivity rate of Wisconsin’s SNT program.

In 2011, Wisconsin was funded by the Health Resources and Services Administration’s Systems Linkages and Access to Care for Populations at High Risk of HIV Infection Initiative. This initiative was a multi-state demonstration project to develop, pilot, and evaluate innovative models of testing and care to improve knowledge of serostatus, linkage to care, retention to care, and viral suppression. As part of this initiative, Wisconsin implemented a protocol that standardized SNT programs across the state. The protocol was developed to ensure the program’s fidelity to CDC’s SNT model, to increase the resources allocated to SNT within each agency, and to increase the positivity rate compared to other CTR programs. This paper describes Wisconsin’s social networks testing program and outcomes.

## Methods

During September 2013 through August 2015 there were 18 sites across Wisconsin that offered CTR testing, of which 12 offered social networks testing and implemented the new protocol. Hereafter, CTR refers to all recruitment methods other than SNT. Six of the 12 SNT sites were in Milwaukee and six were in other areas of the state. The 12 SNT sites represented six agencies, comprised of two AIDS Service Organizations, two community-based organizations, and two federally qualified health centers. The four key elements of the SNT protocol are summarized below.

### Planning and Pre-Implementation

In order to demonstrate readiness to implement the new protocol, agencies were required to conduct planning activities, such as developing staffing plans, identifying populations at risk for HIV infection to be reached by SNT (e.g., transgender women, MSM), and developing a strategy to enlist recruiters from the populations of interest.

### Selection and Support of Recruiters

Recruiter selection included the process of identifying possible recruiters and providing orientation and coaching to those who agreed to participate. Potential recruiters were chosen from recipients of HIV prevention or care services within the agency based on individual characteristics. Agency clients who were representative of or had access to the population of interest, had a positive relationship with the agency and attitude toward HIV testing, and were well-respected within the community were briefed on the program requirements and invited to participate. Program requirements included participating in orientation and coaching sessions with agency staff, recruiting individuals within their own social, sexual, or drug-using networks for HIV testing, and adhering to confidentiality policies. Those who agreed to participate then underwent a program orientation and coaching session, during which recruiters were asked to identify individuals from their networks who matched the population of interest and who might benefit from HIV testing. Recruiters were also coached on techniques for talking with the selected individuals about accessing HIV testing services. Agency staff contacted recruiters one week and one month after recruitment began to check on progress and provide additional coaching if necessary.

### Recruitment and Testing of Network Associates

During the recruitment phase, recruiters referred their NAs for HIV testing. Recruitment was intended to be limited to 20 NAs to ensure that recruiters focused on those most in need of HIV testing. Recruiters could accompany their NAs to the agency for testing, invite an agency staff person to a community location for testing, or refer their NAs to agencies that offered SNT. If the recruiter was not planning to be present when the NA was tested, the recruiter provided the NA with a pocket-card printed with the recruiter’s unique client identifier, which was a combination of the recruiter’s mother’s maiden name and the recruiter’s birthdate. This card was presented at the testing agency in order to link the NA and recruiter in the testing database. Regardless of testing venue, the NA was provided HIV counseling and testing services according to the usual procedures within the agency. Gift card incentives worth $10 were provided to both the recruiter and NA for each HIV testing event.

### Recruiter Transition

The recruiter role was short-term and ended when the recruiter reached the 20 NA cap, could no longer identify members of the desired population to be tested, began to identify NAs who did not represent people at high risk for HIV (e.g., people whom they did not know or who did not report any HIV risk factors), or discontinued voluntary participation.

### HIV Testing and Collection of Individual-Level Data

All sites performed confidential HIV rapid antibody testing followed by a fourth generation diagnostic laboratory algorithm to confirm reactive rapid test results. Individuals were provided risk reduction counseling and linkage to care as necessary. For the purpose of this analysis, positive test results included both reactive rapid test results not confirmed by a laboratory test and laboratory-confirmed positive test results. In order to ascertain whether the positive test result represented a new or previous HIV diagnosis, testing data were matched to the state HIV case surveillance system. Positive tests were considered to represent a new diagnosis if the individual did not have an earlier positive test result documented in the surveillance system. Some positive results were unable to be matched to the surveillance system due to lack of confirmatory testing, anonymous confirmatory testing, or data entry errors; therefore we could not determine whether the positive result represented a new or previous diagnosis.

Agencies collected data associated with the testing event on the screening questionnaire used by all agencies conducting publicly funded HIV testing in Wisconsin. This questionnaire contained fields required by the CDC as part of national HIV monitoring and evaluation standards and fields of local interest, including demographics, behavioral risk factors, the program under which the individual was tested (SNT or CTR), and test results. These data were entered by agency staff into EvaluationWeb^®^, the secure, web-based data system used for collecting and reporting national HIV prevention monitoring and evaluation variables. The unique client identifier for the recruiter was also entered in order to track relationships between recruiters and their NAs.

The unique client identifier was also used to de-duplicate individuals over time for analysis. The first testing event during the study period was used for analysis for individuals with multiple testing events when all events were negative for HIV. The first positive testing event was used for individuals with at least one positive test result, even if the individual had previous negative testing events. The single testing event for each person was then used to categorize individuals by demographics, risk factors, and testing program. If multiple risk behaviors were reported then an overall risk category was calculated according to the hierarchy used by the CDC for HIV surveillance data [10]. MSM included men who reported ever having sexual contact with men or both men and women, excluding those who also injected drugs. Injection drug use (IDU) included people with injection drug use risk (excluding MSM who also injected drugs), and MSM/IDU included those with both MSM and IDU risk. High-risk heterosexual included individuals who reported heterosexual contact with a person known to have, or be at high risk for, HIV infection. High-risk heterosexual excluded men who reported sexual contact with both men and women.

Chi squared tests were used to calculate differences in demographic characteristics and outcomes between SNT and CTR programs. Analyses were performed using SAS^®^ 9.4.

### Qualitative Evaluation

As part of the evaluation, researchers from the Center for AIDS Intervention Research (CAIR) at the Medical College of Wisconsin collected data from participating agencies about the successes and challenges of implementing the protocol in order to provide context to the HIV testing outcomes. DPH staff provided the names and contact information for the supervisors at each of the six agencies conducting SNT. CAIR staff invited each of these individuals to participate in a confidential interview; five supervisors participated in the interview. The interview guide for agency staff included questions regarding how recruiters were chosen, trained, and coached; characteristics associated with successful recruiters; the motivation for NAs to be tested, including the role of incentives; successes and challenges implementing the new protocol; and suggestions for future refinement of SNT. Interviews were transcribed verbatim. The third author read each transcript in its entirety. To distill and describe themes, summaries were written of each transcript, common challenges were noted, and quotes encapsulating themes were selected. Given the scope of this manuscript and the small number of interviews, select findings are presented below to highlight key implementation challenges that may be generalizable to other jurisdictions. Institutional Review Board approval for the qualitative evaluation was obtained from the Medical College of Wisconsin.

## Results

### Participant Characteristics

There were 19,095 tests conducted at the 18 CTR sites during the study period; 1232 individuals tested through SNT and 14,595 tested via CTR. Among individuals participating in SNT programs, 265 recruiters recruited a median of 3 NAs (range 1–63, indicating that some agencies allowed recruiters to exceed the cap of 20 NAs).

Table 1 summarizes the demographic and risk characteristics of individuals tested at the 12 SNT sites during the two-year study period. Approximately 80% of the SNT testers were male; the median age was 29 years. The majority of individuals were non-Hispanic Black (45%) or non-Hispanic White (32%). Based on self-reported risk factors, 47% of SNT participants were categorized as MSM, 9% as MSM/IDU, 25% as IDU, and 10% as high-risk heterosexual. The remaining individuals (8%) were categorized as unknown or other risk (e.g., the individual reported condomless sex with an opposite-gender person but did not meet the definition of high-risk heterosexual). The demographics of SNT compared to CTR participants differed across all demographic categories, with SNT attracting more people who were less than age 24, were Black or who had MSM or IDU risk. In addition, a significantly higher proportion of individuals testing through SNT identified as Black MSM compared to those testing via CTR (28 vs. 8%, p < 0.001). Individuals who received their test through an SNT program were also more likely to report that they were testing for HIV for the first time (35 vs. 27%, p < 0.001).

**Table 1:** Characteristics of individuals tested for HIV at 18 targeted testing sites by testing program, Wisconsin, September 2013–August 2015

	Social networks testing N (%)	Counseling, testing and referral N (%)	P value
Total	1232	14,595	
Gender			P < 0.0001
Female	221 (18)	2749 (19)	
Male	970 (79)	11,706 (80)	
Transgender	40 (3)	132 (1)	
Other	1 (0.08)	8 (0.05)	
Age (years)			P < 0.0001
≤ 24	410 (33)	4237 (29)	
25-34	389 (32)	5150 (35)	
35-44	189 (15)	2436 (17)	
45 and older	244 (20)	2772 (19)	
Race/ethnicity			P < .0001
White	391 (32)	6546 (45)	
Black/African American	556 (45)	5330 (37)	
Hispanic	197 (16)	1916 (13)	
American Indian	39 (3)	215 (1)	
Asian/Pacific Islander	18 (1)	225 (2)	
More than One Race	31 (3)	307 (2)	
Unknown	0 (0)	26 (0.2)	
Risk group			P < .0001
High-risk heterosexual	123 (10)	3765 (26)	
IDU	315 (25)	2069 (14)	
MSM	580 (47)	4955 (34)	
MSM/IDU	113 (9)	276 (2)	
Other risk/unknown	101 (8)	3530 (24)	
Tested positive			
Number of positive results (new positivity rate)	23 (0.49)	137 (0.48)	0.97

*IDU* injection drug use

*MSM* men who have sex with men

### Repeat Testing within Social Networks

Sixty (5%) people tested more than once via SNT (range 2–7 times) and 1913 (13%) people tested more than once through CTR (range 2–16 times). The demographic characteristics of people who tested more than once via SNT are shown in Table 2. Individuals testing more than once were mostly male, Black and had MSM risk. All repeat tests occurred at one of the Milwaukee sites, and all but one individual tested negative at all tests.

**Table 2:** Characteristics of individuals receiving more than once social networks test during the study period

	N (%)
Total	60
Gender	
Female	4 (7)
Male	53 (88)
Age (years)	
≤ 24	26 (43)
25–34	18 (30)
35–44	6 (10)
45 and older	10 (17)
Race/ethnicity	
White	14 (23)
Black/African American	40 (67)
Hispanic	4 (7)
Asian/Pacific Islander	1 (2)
More than one race	1 (2)
Risk group	
High-Risk Heterosexual	4 (7)
IDU	4 (7)
MSM	44 (73)
MSM/IDU	5 (8)
Other risk/unknown	3 (5)

*IDU* injection drug use

*MSM* men who have sex with men

### HIV Testing Results by Strategy

There were 160 positive test results during the study period; 137 from CTR and 23 from SNT. The new positivity rate from SNT tests was 0.49%; 6 tests represented new HIV diagnoses, 8 represented previous diagnoses, and 8 could not be determined. The new positivity rate from CTR was 0.48%; 70 tests represented new HIV diagnoses, 42 represented previous diagnoses, and 25 could not be determined. The majority (88%) of unresolved positive results from both testing programs were preliminary positive results without name-associated confirmatory test results.

### Qualitative Findings

#### Incentives

The use of incentives was a key challenge identified by both DPH staff, and agency staff during the qualitative interview. Agency staff mentioned that recruiters who were primarily motivated by incentives were difficult to enroll or lost interest quickly and disengaged from the process. As one staff member explained, “Most of the time it’s kind of like when they need some money, then that’s an avenue for them to get a couple dollars but if they don’t need any money, they don’t really waste their time trying to recruit people.” Other interview participants talked about the impact of incentives on the NAs, stating that some NAs would test at more than one agency in order to receive multiple incentives. Throughout the study period, some agencies deviated from the protocol and increased the value and type of incentive offered, leading to unequal incentives across the participating agencies.

#### Staff Burden

Some staff members mentioned that the new formalized process of SNT was burdensome relative to the low yield of new HIV diagnoses. Staff members explained that working on SNT was only one aspect of their job on top of several other responsibilities, but with a lot of associated pressure. The burden of running an SNT program affected staff members’ opinions on continuation of SNT: “If this was the only thing that I did and if this was the only thing that people were involved in…, it would probably run a lot more efficiently.”

## Discussion

During the two-year study period, the new HIV positivity rate did not differ from the new positivity rate from CTR programs. This is consistent with other studies showing no significant differences between SNT and other targeted testing programs [9, 11]. Perhaps the most successful aspect of Wisconsin’s SNT then is its ability to reach certain populations, such as Black MSM or people who inject drugs, and individuals testing for the first time. However, the impact of incentives and staff burden should be considered by other jurisdictions developing SNT programs.

While SNT identified a small number of newly diagnosed individuals, many of the positive test results were for previously diagnosed individuals. This is reported in other SNT studies [6, 7, 12], and one author suggests that the efficacy of SNT programs may be overstated given the high proportion of previously diagnosed individuals [13]. There may be many reasons for not disclosing HIV status at the time of testing, including denial of HIV status, desire to link to HIV medical care, receipt of the incentive, or not wanting to disclose to the recruiter [7, 12, 13].

However, many people with negative results also tested multiple times. All 60 of the repeat testers via SNT tested in Milwaukee, of which 15 tested more than once within a one-month period, including one person who tested three times within 20 days. This short timeframe between tests supports the idea of testing for incentives, especially since some Milwaukee agencies offered incentives of higher value. Because SNT had been in existence since 2008, many community members knew how to access SNT without being referred by a recruiter. Alternatively, repeat testing may reflect the same individuals being targeted by multiple recruiters. Milwaukee had an estimated 3055 people living with HIV at the end of 2014, compared to the larger HIV prevalence of cities represented in the landmark SNT studies (New York City 119,095; Seattle 7411; Washington DC 15,867) [10]. The smaller pool of high-risk individuals in Wisconsin may have led to both agencies and recruiters competing for the same individuals, resulting in use of the same recruiters over time, increased incentive amounts, and exceeding the NA cap.

While the outcomes of SNT have been described in the literature, few studies address the resources required to implement a successful program. McCree et al. describe the planning and resources necessary and allude to burden when discussing staff turnover and morale [12]. Gaiter et al. also acknowledge that SNT is more time consuming than other strategies [11]. Staff in Wisconsin discussed the burden of implementing an SNT program in the context of their other prevention work. Finding, coaching, and supporting recruiters is time-consuming in comparison to other CTR recruitment strategies, and may not seem worthwhile when the new positivity rate is low. In fact, SNT studies in the published literature have been conducted in large, relatively high HIV prevalence jurisdictions, and cost-effectiveness analyses acknowledge that the cost-utility of SNT is variable depending on the number of HIV diagnoses identified [14] and HIV prevalence [8]. In addition, staff comments about burden may reflect burnout and frustration over new rules for a program that had been in existence for 5 years.

## Conclusion

Social networks testing in Wisconsin detected a small number of new HIV infections, although further success may have been hindered by implementation challenges coupled with the oversaturation of SNT sites in a small geographic area. However, due to its ability to reach populations most affected by HIV and individuals not previously tested for HIV, SNT should be considered as one of several recruitment methods. Additional research is needed to determine the effectiveness of SNT in smaller cities with lower HIV prevalence.
